# Exploring Australian News Media Portrayals of Sustainable and Plant-Based Diets

**DOI:** 10.3390/nu16070996

**Published:** 2024-03-28

**Authors:** Rimante Ronto, Carla Vanessa Alves Lopes, Diana Bogueva, Barbara Davis, Alexandra J. Bhatti, Priscilla Navarrete, Josephine Y. Chau

**Affiliations:** 1 Department of Health Sciences, Faculty of Medicine Health and Human Sciences, Macquarie University, Macquarie Park, NSW 2109, Australia; 2 Curtin University Sustainability Policy Institute (CUSP), Curtin University, Perth, WA 6102, Australia

**Keywords:** sustainable diets, sustainability, media coverage, nutrition, Australia

## Abstract

(1) Background: Dietary behaviour transformation is imperative for the attainment of more sustainable food systems, including an increased intake of plant-based foods and lower consumption of red meat and highly processed foods. The influence of news media coverage on public opinion regarding dietary behaviours is significant. Therefore, this study aimed to explore how sustainable/plant-based diets have been portrayed in Australian news media. (2) Methods: The Factiva global news database was used to search news articles published in Australia between 2018 and 2020. Relevant news articles were selected if they included keywords relating to sustainable diets, plant-based diets, and meat alternatives. We used a coding protocol to extract key information, such as date of publication, article topic, and any health, environmental and economic impacts. Then, we performed a framing and thematic analysis of the data. (3) Results: From 357 included articles, more than half of the articles encouraged increasing the intake of plant-based foods (53.5%) and reducing animal-derived food intake (55.2%). Several reasons for such shift from animal protein centric Australian diets were identified throughout the articles such as health benefits (15.4%), environmental impacts (11.2%), animal welfare (4.8%), seasonality and local food intake (5.3%), avoiding overconsumption (4.5%) and food wastage (4.5%). (4) Conclusions: The predominant frame in Australian news coverage about sustainable diets has been about consumption, more plant- and less animal-based products, with little nuance about the complex interplay of diet quality and environment in influencing food choices. Australian news media should broaden its coverage of sustainable diets to include health, environmental, and economic factors to improve public understanding and facilitate informed and sustainable food choices. Further research is needed to enhance comprehension of how the audience perceives media coverage on this topic, which will provide a more thorough understanding.

## 1. Introduction

The current global food systems are not environmentally sustainable and are major causes of depleting non-renewable resources [1], including approximately 70% of freshwater depletion [2,3,4], up to 30% of green gas emissions [5,6] and approximately 40% of global arable land depletion [2,7]. In Australia, extreme weather events resulting from climate change have impacted the food supply chain with crop losses, a rise in food prices and, consequently, a direct impact on food security [8].

Current dietary behaviours have significantly contributed to the burden of obesity and noncommunicable diseases (NCDs) such as diabetes, stroke, coronary heart diseases, and cancer [9]. Globally, 11 million deaths were attributed to dietary risk factors in 2017 [10]. In Australia, it was estimated that 66.4% of Australian adults in 2017–2018 [11] and 65.8% in 2022 [12] were overweight or obese, and almost half of Australians (47.3%) had one or more chronic conditions [11]. In 2022, overweight and obesity rank as the second leading contributor to poor health and early mortality in Australia, and they also adversely affect both physical and mental health and overall wellbeing, underscoring the need for effective interventions [13].

To address the burden of chronic diseases and negative environmental consequences of unhealthy diets, diets that are both healthy and environmentally friendly are needed. The Food and Agriculture Organization (FAO) of the United Nations (UNs) described sustainable and healthy diets as “*those diets with low environmental impacts which contribute to food and nutrition security and to healthy life for present and future generations*” [14]. Studies have suggested reducing the consumption of animal-derived foods such as red meat and discretionary, ultra-processed foods and increasing the consumption of more plant-based foods, as essential elements for significant food system changes [15,16]. It is important to note that ‘sustainable diets’ and ‘plant-based diets’ are often used interchangeably in the literature. For over two decades, the term ‘plant-based diets’ has been used to describe some dietary patterns. However, this term has no universally agreed-upon definition [17]. Plant-based eating encompasses a spectrum of dietary approaches, ranging from the exclusion of all animal products to the inclusion of small-to-moderate amounts of animal products [16]. Some definitions of ‘plant-based diet’ refer to diets with a low content of animal-source foods, such as meat, fish, eggs and dairy products, and a high content of vegetables, fruits, legumes, seeds, and nuts [17]. Others are more precise and refer to vegetarian (lacto-ovo-vegetarian) and vegan diets (excluding all animal-source foods), as the portions of the different food groups are not defined [17]. However, a sustainable diet is a broader concept; it also considers environmental aspects of plant-based foods (e.g., how these foods are grown, processed, and distributed). An example of this distinction is seen in the sourcing of produce: a plant-based diet may include imported fruits and vegetables, but a sustainable diet considers the entire food production life cycle and would prioritize locally grown, organic produce to reduce the carbon footprint and support local farmers.

There is robust evidence of the beneficial effects of plant-based foods on health, including weight status and systemic inflammation [18]. While Western diets, which include highly processed foods, meats and sweetened beverages, are associated with insulin resistance and type 2 diabetes, plant-based foods can promote better blood parameters such as decreased blood glucose levels, total cholesterol and LDL cholesterol [19,20]. A high fibre intake from vegetables, fruits and whole grains may be one of the potential biological pathways that affect these parameters [20]. Smith and Tucker [21] have shown that a 2–10 g/day increase in soluble fibre intake resulted in a significant decrease in LDL cholesterol, likely linked to lower cholesterol and fat absorption. Shifting to a more sustainable diet high in plant intake is likely to lessen the impact on the environment by reducing up to 50% of greenhouse gas emissions and 62% of land use [22].

A systematic review and meta-analysis of twelve cohort studies with 508,861 participants demonstrated the potential protective role of diets rich in plant-based foods against cardiovascular disease, coronary heart disease, cerebrovascular disease and cancer mortality [23]. More specifically, the authors showed an inverse association between plant-based diets and the risk of all-cause mortality (reduction of 8%), with a hazard ratio of 0.92 for a healthy plant-based diet, 0.81 for a pesco-vegetarian diet, and 0.74 for a pro-vegetarian diet [23]. In the EPIC-Oxford cohort study, which examined the association of a vegetarian diet with the risk of incident ischemic heart disease, researchers found that after 11 years of follow-up, vegetarians had a 32% lower risk of incident ischemic disease than nonvegetarians [24]. Furthermore, evidence from RCTs demonstrate benefits to cardiometabolic risk indicators, such as body mass index, fasting glucose, and LDL cholesterol, of plant-based diets compared with omnivorous diets [25,26].

Although the evidence shows that sustainable diets are associated with better environmental and health outcomes [15,16], and the Australian Dietary Guidelines promote healthy diets [27], the 2017–2018 Australian National Health Survey [11] showed that only 5.4% of adults met both fruit and vegetable recommendations. On the other hand, in 2011–2012, Australians aged 19 years and older exceeded the total energy intake and the recommendations for red meat per week [28]. Furthermore, 35% of Australian total energy intake was from discretionary foods, considered high in sugar, salt and saturated fats [28].

To shift the current epidemiological and environmental scenario, healthy and environmentally sustainable diets are needed.

In recent times, there has been a noticeable surge in media coverage discussing sustainable diets [29]. This increased media attention on diet and nutrition can wield a significant impact on public opinions and dietary behaviours [30]. The media, deeply integrated into various aspects of society, plays a pivotal role in driving social and cultural changes [31,32]. With mass media platforms such as television, newspapers, and online sources reaching a wide audience, there exists immense potential to influence public views and mould health-related behaviours on various topics [31,32,33,34,35,36,37]. As an example, news media coverage of diet and nutrition issues can significantly shape public opinion in a specific direction, yielding both positive and negative impacts on public health.

Freisling et al. [38] conducted a cross-sectional nutrition survey and showed that newspaper articles, the internet, and booklets were sources of nutrition information and were associated with daily fruit and vegetable consumption among adolescents. Interestingly, the authors found that exposure to radio commercials had a negative impact on fruit and vegetable consumption. Another study evaluated the health content of beverage-related news reports on national television newscasts, where the authors found confusing and conflicting nutrition recommendations in 29% of the reports [39].

The pervasive influence of misinformation disseminated through news media has profound implications for public health. Inaccurate or misleading health information can fuel vaccine hesitancy and lead to incorrect interpretations of scientific evidence as well as have impacts on mental health and diet behaviours [40,41,42]. False narratives surrounding health may undermine trust in healthcare institutions and health guidelines, impeding effective public health interventions [40]. Economic loss, loss of health and lives, and loss of institutional reputation are some of the significant effects of misinformation on individuals and society [43]. Recognising the potential significance of news media as a critical communicator for health behaviour change, studies have been reporting how some crucial epidemiological topics are portrayed through different media channels. These topics include diet-related health issues [44], mental illness [45,46], COVID-19 [47] and general public health [48]. Therefore, it is important to explore how sustainable/plant-based diets have been portrayed in Australian news media. Specifically, the study aimed to analyse: (1) the nature of news coverage of sustainable/plant-based diets; (2) which aspects of sustainable/plant-based diets attract news media attention; (3) how the relationship between sustainable/plant-based diets and health, environment, and economy is framed; and (4) the overall orientation of newspaper articles towards sustainable/plant-based diets.

## 2. Materials and Methods

The Factiva global news database was searched for online news articles published in Australia between 2018 and 2020 (Syntax: “sustainable diet” and “plant-based diet”; region: Australia; date of search: 19 November 2020; with duplicates removed). We used terms ‘sustainable diet’ and ‘plant-based diet’ as often these terms are used interchangeably in the literature. Moreover, including both terms allows for a more holistic and nuanced approach to discussing how news media portrays plant-based diets and sustainable diets as linked to sustainability. Plant-based diets centre around food sources primarily derived from plants, whereas sustainable diets go beyond just the source of food. They consider the entire food production life cycle, taking into account environmental impact, social aspects, and economic benefits. The EAT-Lancet Commission report [15] on healthy diets from sustainable food systems was published in early 2019 attracting a lot of media attention, therefore we aimed to capture this sampling frame. Relevant news articles featuring the following keywords in the headline or first three paragraphs were included in this review: ‘sustainable diet’, ‘plant based diet’, ‘plant proteins’, ‘meat substitute’, ‘meat alternative’, ‘meatless’, ‘fake meat’, ‘lab grown meat’ and ‘clean meat’.

A coding protocol based on previous studies [48,49] was used to extract key information from each newspaper article which included: item ID number, date of publication, name of newspaper, page number, by-line, article headline, article word count, section of publication, main topic of first paragraph, news angle, any individuals or groups mentioned in the article, all sentences including the phrase “sustainable diet” and/or “plant based diet”, list of sustainable diet components discussed in the article, all sentences discussing sustainable/plant-based diet and either health, environment or economy, and anything else of interest (Table 1).

Six investigators (RR, DB, BD, AJB, PN and JYC) independently extracted data from the included studies. The Principal Investigator (RR) additionally cross-checked data extraction sheets from all investigators (20% of total number of articles), with any disagreements in extraction and coding resolved through the discussion. Framing and thematic analyses were performed. All sentences which included the term ‘sustainable OR plant-based diet’ were captured. Coding was conducted by one researcher (CVAL), allocation of framings to groups was discussed and confirmed by another researcher (RR) and approved by all co-authors. An investigator triangulation technique was used to validate qualitative data and its interpretation [50,51]. The data analysis was performed by a research team consisting of professionals from different disciplines, including public health, nutrition and dietetics, and environmental health. Triangulation helped to prevent the personal or disciplinary biases of a single investigator from excessively influencing the findings [50,51].

## 3. Results

An initial search for the term ‘sustainable diet’ generated only 13 articles as the term was relatively new. Therefore, we performed a second search using the broader term ‘plant-based diet’. The new search generated an additional 506 articles. The final sample included 357 articles after removing duplicates and excluding articles such as advertisements and recipes (*n* = 148 in 2018, *n* = 194 in 2019, *n* = 15 in 2020).

**Figure 1:**
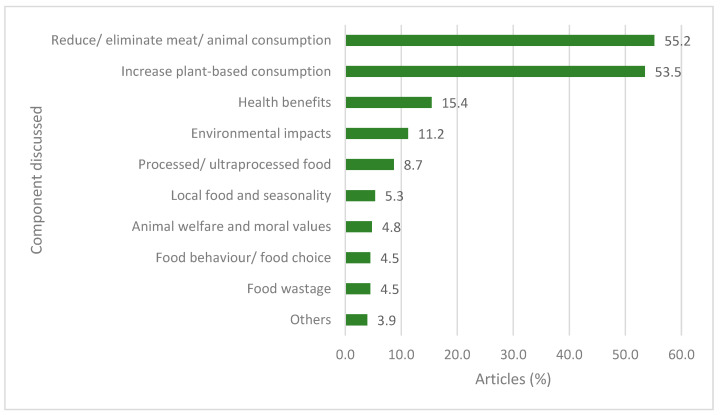
Components of sustainable and healthy diet discussed in Australian news media between 2018 and 2020 (*n* = 357).

The main components discussed in the articles about sustainable and healthy diets in Australian news media are summarised in Figure 1. More than half of the articles encouraged the reduction of elimination of animal-derived food intake (55.2%) and advocated for increased intake of plant-based foods (53.5%). One third of the articles discussed reasons for such shifts in current diets such as benefits for health (15.4%), environmental impacts (11.2%), and animal welfare and moral values (4.8%). However, around 9% of the articles expressed concerns about consuming highly processed plant-based foods. These concerns were due to the increased number of vegan products, which may be energy-dense, rich in fat, and have added sugar, salt, and artificial ingredients. The articles also discussed the importance of seasonality and local food intake (5.3%), avoiding overconsumption (4.5%) and food wastage (4.5%) to achieve a more sustainable and healthy diet.

Two major themes were formed from the analysis of articles discussing sustainable and healthy diets: (1) Food choices and behaviours related to sustainable and healthy diet; and (2) Social, environmental, and economic reasons for and impacts of sustainable and healthy diet. The themes, subthemes and illustrative quotes are described in detail below.

### 3.1. Food Choices and Behaviours Related to Sustainable Diets

#### 3.1.1. More Plant-Based Foods and Less Animal-Derived and Ultra-Processed Foods

The articles related sustainable and healthy diets by discussing increasing minimally processed plant-based foods such as fruits, vegetables, legumes, and nuts and reducing or eliminating animal-derived and ultra-processed foods. This type of diet has been described as healthier, more nutritious, and environmentally friendly.

*“When you’re eating a plant-based diet that’s full of wholefoods, like fruits, vegetables, grains, nuts, legumes, this is obviously healthier”*

(*Australian Broadcasting Corporation News, 11 September 2019*)

*“Healthy and sustainable dietary recommendations promote the consumption of fewer processed foods, which are energy-dense, highly processed and packaged”*

(*The Newcastle Herald, 3 January 2020*)

*“So, instead of having meat or chicken at every meal, replace it with some plant-based alternatives like legumes or tofu two or three times a week”*

(*The Newcastle Herald, 3 January 2020*)

#### 3.1.2. Buying Local Foods and Reducing Food Wastage Are Important

Several articles indicated the significance of reducing food wastage and adopting a locally sourced diet as crucial component to achieve a sustainable and healthy lifestyle. The articles mentioned avoiding food wastage and eating locally produced foods not only have a positive impact on the environment by mitigating related/associated greenhouse gas emissions, but also deemed indispensable in addressing the dietary needs of a growing global population.

*“[…] transitioning towards a more sustainable diet involved eating more locally produced foods, less processed foods (particularly those made from many different ingredients) and reducing food waste”*

(*Stock & Land, 27 October 2018*)

#### 3.1.3. It Is Hard but Possible

Some articles stated that it is not necessary to remove all animal derived foods from diets and that it may not be easy to follow a sustainable and healthy diet, whilst listing some suggestions to help those who want to follow this dietary pattern. The main suggestion was to take a slow approach in reducing animal-derived foods. Other advice included changes to food preparation and cooking skills such as diversifying meal preparation and recipes to help with an increase of plant-based foods with respecting people’s preferences, traditions, and cultures.

*“Ms Abraham said this was by substituting a few meals a week for plant-based alternatives, rather than deferring to meat proteins”*

(*The Advertiser, 20 October 2019*)

*“Plant-based diets can be adapted to suit your taste preferences, traditions and cultures…”*

(*The Conversation, 5 July 2019*)

### 3.2. Social, Environmental and Economic Reasons for and Impacts of Sustainable and Healthy Diets

#### 3.2.1. Health Benefits of Following Sustainable and Healthy Diets

The articles reported on health benefits of following a sustainable and healthy diet such as improved gut health, prevention of chronic diseases (diabetes, heart diseases, cancer), reduced inflammation and pain, sport performance enhancement, weight loss, improved mental health and reducing overall mortality.

*“…a predominantly whole-food plant-based diet with an abundance of diversity is a great way to optimise gut health”*

(*Mail Online, 30 July 2019*)

*“They read last week’s study revealing a plant-based diet could reduce the risk of developing Type 2 diabetes and significantly improve mental health”*

(*Courier Mail, 11 November 2018*)

In contrast, some of the articles stated that a plant-based diet can drive some health risks due to nutrient deficiency or due to vegan ultra-processed food options. Also, some of these articles stated that there might be no health benefits at all. They suggested including supplements to achieve a balanced diet for those who follow a plant-based diet.

*“But while a plant-based diet can have many health benefits, eliminating certain food groups can put you at risk of becoming nutrient deficient”*

(*Mail Online, 2 July 2018*)

#### 3.2.2. Positive Environmental Impacts of Following Sustainable Diets

In general, the articles support that a sustainable and healthy diet has positive impacts on the environment and climate change, resulting in reduced greenhouse emissions, plastic production and usage, and land use.

*“I switched to a plant-based diet two years ago to reduce my carbon footprint and have drastically cut my single use plastic consumption”*

(*St George and Sutherland Shire Leader, 26 September 2019*)

*“An upcoming report is likely to provide the scientific basis of calls for people to switch to a plant-based diet to combat climate change”*

(*The Strait Times, 7 August 2019*)

Conversely, some articles stated that adopting plant-based diets does not necessarily equate to enhanced sustainability. These articles argued that increasing the intake of plant-based foods could negatively impact the environment through an increase in carbon footprint, chemical use, and a surge in deforestation.

*“Reducing animal-based products in favour of plant-based products did not impact the environment any less as the carbon footprint was not greatly reduced”*

(*Stock & Land, 28 October 2018*)

#### 3.2.3. Animal Welfare

Some articles provided testimonials from consumers who adopted a diet which does not include animal products outlining their reasons and motivations for doing so, notably including the concerns about animal cruelty and suffering.

*“A key reason people adopt a plant-based diet is concerns about animal cruelty and suffering”*

(*LoneWolfFilmsNZ.com, 5 November 2018*)

On the other hand, a divergent perspective is presented in two other articles regarding the relationship between plant-based diets and animal welfare. One article stated that a plant-based diet still results in the death of thousands of animals where the other article supported the benefits of livestock, emphasising the drawbacks of a complete shift to a plant-based diet. This article underscores the multifaceted benefits of livestock, such as their deployment on unsuitable crop production land, contribution to livelihoods, and the various advantages animals offer.

*“But those who opt for a plant-based diet may be surprised to learn that their lifestyle still results in the death of hundreds of thousands of animals each year”*

(*Mail Online, 26 June 2019*)

*“A shift towards a radically plant-based planetary diet loses the many benefits of livestock—including its deployment on land that is not suitable for crop production, its contribution to livelihoods, and the many other benefits that animals provide.”*

(*The Conversation, 16 January 2019*)

#### 3.2.4. Economic Impact of Sustainable and Healthy Diets

Some articles discussed a growing market as well as the proliferation of plant-based foods available in supermarkets. As well as a rise in plant-based diet followers in the last decades due to information on social media, documentaries, blogs, and the sharing of famous people’s routines who have adopted a plant-based diet. The articles discussed the increased number of followers on blogs and social networks who promote plant-based diets including influencers and celebrities, the popularity of documentaries that addressed this topic, and the record number of vegan products that have been sold.

*“The report showed a “positive outlook” for the industry, indicating the next decade would see growth in plant protein consumption because of consumer demand for healthy, sustainable and affordable food”*

(*The Weekly Times, 23 October 2019*)

*“The rise of the vegan trend is being fuelled through social media communities that continuously promote a plant-based diet by posting colourful and aesthetically pleasing food photos.”*

(*Mail Online 26 September 2018*)

As a result of the increased number of consumers of plant-based diets, some articles indicated the importance of plant-based food market and products for the economy and how this is an area of growth in Australia. In addition, some articles emphasised possible positive economic gains due to healthcare related savings and positive impact on climate change if the majority of people were to adopt a sustainable and healthy dietary pattern.

*“We’re well and truly aboard the plant-based diet craze—it’s pegged to beef up the Australian economy by $3 billion by 2030—but there’s a new protein crawling onto plates around the country”*

(*The New Daily, 9 January 2020*)

*“The Vegan Society calculates that a worldwide shift to plant-based diets could result in healthcare-related savings and avoided climate damages worth $1.5tn”*

(*The Newcastle Herald, 3 January 2020*)

Even though some articles emphasised possible positive economic impacts of sustainable and healthy diets, some articles indicated the economic limitations of such a practice. Two articles were sceptical about the feasibility of the entire population adopting a sustainable and healthy diet and mentioned concerns about the feasibility of feeding Australia’s population solely with plant-based foods.

*“Their call to arms is shaky on a number of levels, so let’s explore the practicality if everyone stops eating meat and see if we can feed Australia on plant-based diet”*

(*The Land, 15 August 2019*)

## 4. Discussion

The news media plays an important role in influencing public opinion and behaviour about health and diet. This exploration of narratives of how sustainable and plant-based diets were presented in the Australian news media revealed several health, environmental and economic benefits of a sustainable diet, mirroring important topics presented by the UN’s FAO concept and framework for sustainability in food systems [52] and the Sustainable Healthy Diets: Guiding Principles [53]. Including support for a variety of minimally processed foods such as fruits, vegetables and whole grains, restricting highly processed foods and red meat, avoiding overconsumption, maintaining greenhouse gas emissions and use of natural resources within set targets, and reducing food loss and waste. Interestingly when considering the Sustainable Healthy Diets: Guiding Principles [53], some important aspects were not identified as being discussed in the media in this analysis, including accessible foods and affordability.

A similar study in Romania in 2014–2017 identified 314 articles with unclear and general coverage of sustainable food in the media [54]. The authors found the four main topics related to sustainable food were nutrition, food related to a specific health condition, food in general and diets. Also, only a few articles (*n* = 17) covered sustainable food production, including junk foods, organic crops and national standards and labels related to food [54]. These results could be due to a lack of conceptual clarification for sustainable diets until early 2019 when the EAT-Lancet Commission published its report [15], and the UN’s FAO and World Health Organization (WHO) published the Sustainable Healthy Diets: Guiding Principles [53].

Most articles (~52%) in this study discussed the reduction of animal source consumption and the increase of plant-based foods as part of a sustainable diet. Similar to these findings, a study in the United Kingdom (UK) found that most online news sites included the narrative that meat negatively impacts the environment, with around 60% of the articles recommending eating less meat [55]. In contrast, Sievert et al. [56], analysed frames used by interest groups in relation to red and processed meat by reviewing 150 news media articles in the USA, UK, Australia and New Zealand. They categorized approximately 40% of the articles as ‘pro-meat’, 35% as ‘pro-reduction’ and 21% as ‘neutral’ [56]. The authors highlighted that the interpretation and portrayal of red and processed meat in the news media was influenced by groups with different interests, such as academics, the meat industry and policymakers [56].

National and international dietary guidelines and studies show less health and environmental negative impacts of a diet with high plant-based foods and low in red and processed meat [15,16,27,53]. Gibbs and Cappuccio [57] demonstrated in their review that transitioning to a plant-based diet could reduce diet-related greenhouse gas emissions by 49%, land use by 76% and green and blue water use by 21%. Also, a diet low in animal products and high in plant-based food can reduce the burden of obesity and chronic diseases [57]. A multicentre prospective cohort study in Europe, using data from 443,991 participants, estimated that higher adherence to a sustainable diet could potentially reduce up to 39% of cancers, 63% of deaths, 50% of greenhouse gas emissions and 62% of land use [22]. Although there is still a lot remaining to explore, studies have shown that transitioning to a plant-based diet may also contribute to increasing the diversity of health-promoting bacteria in the gut [58], reducing the pathobionts and increasing the protective species [59], and improving some metabolic parameters such as lipid profile, oxidative balance and glucose homeostasis [60].

The literature also confirms the economic benefits that Australian news media articles mentioned, including the increased number of plant-based food consumers and products. One study showed that from 2015 to 2019, there was a 429% increase in plant-based meat substitute products in four metropolitan Sydney supermarkets, reaching 614% specifically for burgers [61]. However, a challenge to following a plant-based diet identified in this study was the nutritional composition of ultra-processed plant-based foods, which is also present in the literature, mainly concerning saturated fat, sugar, and salt [62]. Vellinga et al. [63] assessed the nutritional composition of plant-based burgers and reported high energy levels, sodium and total fats. An audit of plant-based meat substitutes on supermarket shelves in Sydney supermarkets collected data from 137 products and showed that sodium is a public health issue for these products [61]. The authors found approximately six times more sodium in plant-based meat mince than in meat mince [61]. Similarly, increased consumption of plant-based alternatives presents potential food safety and health risks. These include concerns about nutritional adequacy, encompassing aspects such as digestibility and inaccessibility, as well as worries regarding allergenicity and toxicity [64].

Findings from this study also identified a narrative that plant-based diets can be nutrient deficient. However, studies have shown nutrient inadequacies across all dietary patterns [65,66,67]. A 2021 systematic review, including 141 studies, compared the nutrient intake and status of adults consuming plant-based diets and meat eaters [65]. The results demonstrated that a plant-based diet could increase the risk of inadequate nutrient intake of some nutrients (calcium, iron, zinc, iodine, EPA/DHA, vitamin B12, D), but could also improve the intake of fibre, polyunsaturated fatty acids (PUFA), α-linolenic acid (ALA), folate, magnesium and vitamin E. The study also showed that meat-eaters could be at risk of inadequacy for fibre, vitamins D, and E, folate, magnesium, and calcium [65]. A Spanish cohort study with more than 17,000 participants found that a diet with a lower environmental impact has more dietary fibre and lower levels of saturated fats, sodium, vitamin B12, zinc and calcium [60]. Craig et al. [66] state that when appropriately planned, a plant-based diet is safe and effective for all ages and life cycles, including athletes, pregnancy and lactation stages. The authors emphasize the importance of health professional support, dietary guidelines and strategies such as fortified foods, supplements and nutritional education [66].

## 5. Strengths and Limitations

This study exhibits both strengths and limitations. One notable strength of this study lies in the use of the Factiva electronic news database for data collection which resulted in facilitation of a comprehensive collection of articles from all Australian news outlets for thorough analysis. Additionally, the study benefits from the analysis of data spanning a three-year period. However, a limitation of this study is that the chosen search terms used may have not captured all references to sustainable diets in the news media throughout the specified time frame, despite the authors conscientious efforts to employ a number of different search terms. In addition, the study acknowledges that the majority of the news articles reviewed concentrated on only one or two specific components of sustainable diets and their associated benefits. Using the term ‘plant-based diet’ in the initial search may have led to most of the included articles being about eating more plants and less animal products. This may limit the overall comprehensiveness and representation of the broader topic. Also, while emphasising the importance of public health media advocates informing the public about the complex interplay between diet and the environment, the study may itself simplify this intricate relationship in its analysis, potentially overlooking nuanced aspects. While recognizing the need for further research, the study does not provide insights into the potential impact of media coverage on public perceptions and behaviours, leaving a gap in understanding the real-world implications of the analysed media articles.

Another limitation is that this study is specific to Australian mainstream news only and does not include social media or news in languages other than English. Research in other countries and with non-English media outlets may yield different insights. Given the global nature of this issue, future similar studies in other countries are warranted.

## 6. Conclusions

This study has explored media reporting of sustainable diets in the Australian online news media using a framing and thematic analysis. The main components discussed were the reduction of animal consumption, the increase in plant-based food consumption, and the health and environmental benefits of plant-based diets. The themes around the narratives also included the social and economic impacts of plant-based diets and the importance of local foods and reducing food wastage.

To our knowledge, this is the first study in Australia that described the news media coverage of sustainable diets. Further research is needed to better understand the influence of news media on sustainable diet behaviours among the Australian population. The call for further research to enhance comprehension of how the audience perceives media coverage on sustainable diets is needed to delve into audience perspectives and to provide comprehensive understanding with audience feedback.

## Figures and Tables

**Table 1:** Coding protocol for news articles featuring the term sustainable/plant-based diet.

Item	Field Name (Description)
1	Item ID number
2	Date of publication
3	Source
4	Page number
5	Byline (Who wrote this article?)
6	Article headline
7	Article word count
8	Section of publication
9	Main topic of first paragraph (What is the issue being reported/discussed? E.g., new diet, celebrity, veganism etc.)
10	News angle (What aspect of the issue/topic attracted the journalist(s)?)
11	Individual(s) or group(s) mentioned in the article (if any)
12	All sentences including the phrase ‘sustainable diet’ and ‘plant-based diet’ for frame analysis
13	Sustainable/plant-based diet component(s) discussed (e.g., Processed food/meat intake, meat reduction, food wastage, fruit and vegetable intake etc.)
14	All sentences including phrases on sustainable/plant-based diet and health impact
15	All sentences including phrases on sustainable/plant-based diet and environment impact
16	All sentences including phrases on sustainable/plant-based diet and economic/financial impact
17	Resources used in the article (e.g., Lancet report, research articles, policy documents etc.)
18	Anything else of interest?

## Data Availability

The data presented in this study are available on request from the corresponding author due to the size of files.
